# Annual acknowledgement of reviewers

**DOI:** 10.1186/1471-2199-15-2

**Published:** 2014-01-28

**Authors:** Tim Sands

**Affiliations:** 1 BioMed Central, Floor 6, 236 Gray's Inn Road, London WC1X 8HB, UK

## Abstract

**Contributing reviewers:**

The editors of BMC Molecular Biology would like to thank all our reviewers who have contributed to the journal in Volume 14 (2013).

## 

Jinwoo Ahn

USA

Maria Ines Almeida

USA

Paola Amable

Brazil

Bogi Andersen

USA

Veronique Baron

USA

Idriss Bennani-Baiti

Austria

Albrecht Bindereif

Germany

Atle Bones

Norway

Gloria Borgstahl

USA

Marco António Campinho

Portugal

Maria Ciaramella

Italy

Fabio Ciccarone

Italy

Amelia Cimmino

Italy

Liza Cubeddu

Australia

Carlos De Noronha

USA

Xiangqun Deng

China

Rao Desirazu

India

Jose Die

USA

Isabelle Duluc

France

Manuela Ferracin

Italy

Trine Fink

Denmark

Felix Freuler

Switzerland

Ulrich Frey

Germany

Shailendra Giri

USA

Mark Glover

Canada

Manfred Gossen

Germany

Steven Gregory

USA

Thomas Greither

Germany

Jiannan Guo

USA

Jason Hinds

UK

Isobella Honeyborne

UK

Konrad Huppi

USA

Thomas Jensen

Denmark

Fatah Kashanchi

USA

Hiroshi Kataoka

Japan

Kristopher Kilian

USA

Bernd Knoll

Germany

Wilfried Kues

Germany

James Lister

USA

David Lombard

USA

Jirong Long

USA

Kristen Lynch

USA

Sam Martin

UK

Peter Messiaen

Belgium

Dirk Motzkus

Germany

Takashi Nakatsuka

Japan

Greg Oakley

USA

Michael O'Connor

USA

Nicolas Paquet

Australia

Jae-Il Park

USA

Jose Manuel Pérez Cañadillas

Spain

Vlad Popovici

Czech Republic

Barry Powell

Australia

Yitao Qi

USA

Isabelle Richard

France

Gareth Roberts

UK

Tom Savage

USA

Aaron Schetter

USA

Michael Schwarz

Germany

Michael Sehorn

USA

Joann Sekiguchi

USA

Eric Sibley

USA

Daniel Starczynowski

USA

Masataka Sugimoto

Japan

Mark Szczelkun

UK

Gerald Thiel

Germany

Bo Thomsen

Denmark

Michal Toborek

USA

Luisa Trindade

Netherlands

Jozef Vanden Broeck

Belgium

Ivan Vannini

Italy

Jesper Troelsen

UK

Pierre-Olivier Vidalain

France

Sonja Maria Walzer

Austria

Guliang Wang

USA

Gregory Weber

USA

Michael Wyatt

USA

Juanjuan Xiang

China

Weidong Xiao

USA

Wang Xicai

China

Brian Yard

USA

Je-Hyun Yoon

USA

Nagahama Yoshitaka

Japan

Helong Zhang

China

Lemin Zheng

China

